# Effects of boxing interventions on physical fitness and health-related quality of life in older people with Parkinson’s disease: a systematic review with meta-analysis

**DOI:** 10.3389/fpubh.2025.1589512

**Published:** 2025-06-06

**Authors:** Jordan Hernandez-Martinez, Izham Cid-Calfucura, Edgar Vázquez-Carrasco, Nicole Fritz-Silva, Tomás Herrera-Valenzuela, Braulio Henrique Magnani Branco, José Zapata-Bastias, Pablo Valdés-Badilla

**Affiliations:** ^1^Department of Physical Activity Sciences, Universidad de Los Lagos, Osorno, Chile; ^2^Department of Education, Faculty of Humanities, Universidad de la Serena, La Serena, Chile; ^3^Department of Physical Activity, Sports and Health Sciences, Faculty of Medical Sciences, Universidad de Santiago de Chile (USACH), Santiago, Chile; ^4^Occupational Therapy School, Faculty of Psychology, Universidad de Talca, Talca, Chile; ^5^Centro de Investigación en Ciencias Cognitivas, Faculty of Psychology, Universidad de Talca, Talca, Chile; ^6^VITALIS Longevity Center, Universidad de Talca, Talca, Chile; ^7^Department of Health, Universidad de Los Lagos, Puerto Montt, Chile; ^8^Graduate Program in Health Promotion, Cesumar University (UniCesumar), Maringá, Brazil; ^9^Sports Coach Career, School of Education, Universidad Viña del Mar, Viña del Mar, Chile; ^10^Department of Physical Activity Sciences, Faculty of Education Sciences, Universidad Católica del Maule, Talca, Chile

**Keywords:** neurodegenerative diseases, dopamine, postural balance, aged, physical functional performance

## Abstract

**Objective:**

This systematic review with meta-analysis aimed to evaluate the available body of published peer-reviewed studies on the effects of boxing (BOX) interventions on balance, cardiorespiratory fitness, motor function, and health-related quality of life (HRQoL) in older people with Parkinson’s disease (PD).

**Methods:**

A comprehensive search of the literature, including peer-reviewed randomized and non-randomized controlled trials, was conducted to December 2024 in the databases of PubMed, Medline, Psychology and Behavioral Sciences Collection (EBSCO), CINAHL Complete, Scopus, and Web of Science (core collection). A random-effects model was employed, and Hedge’s g effect sizes (ES) were computed. The GRADE, RoB 2, ROBIN-1, TESTEX, and PRISMA tools evaluated the methodological quality and certainty of evidence. The protocol (code: CRD42024614097) was registered in PROSPERO.

**Results:**

Eight studies were included, with 100 older people with PD, of which only three could be meta-analyzed. No significant effects were evident (*p* = 0.05), which were small to moderate effects of BOX on ABC-Scale (ES = −0.56; *p* = 0.13), Timed Up-And-Go (TUG; ES = 0.24; *p* = 0.34), TUG dual task (ES = 0.20; *p* = 0.41), 6-min walking test (ES = 2.16; *p* = 0.23), and PD Quality of Life Questionnaire (ES = −0.009; *p* = 0.98).

**Conclusion:**

BOX interventions do not significantly improve balance, cardiorespiratory fitness, and health-related quality of life in older people with PD.

## 1 Introduction

Parkinson’s disease (PD) is characterized by the loss of dopaminergic neurons in the substantia nigra pars compacta and the accumulation of Lewy bodies (1); as the disease progresses, spinal structures, limbic system, nucleus accumbens circuitry, forebrain, and neocortex are affected (1). Cardinal motor symptoms of PD include tremors, rigidity, bradykinesia/akinesia, and postural instability, which slow movement, cause tremors at rest, and affect posture and gait (2), impacting motor function in middle-aged and older people (3, 4), altering motor control (3), balance (5), cardiorespiratory fitness (6), increasing the risk of functional dependence (7), affecting their health-related quality of life (HRQoL) (7).

Therefore, it is important to implement interventions, such as physical activity that improves balance, cardiorespiratory fitness, motor function, and HRQoL in older people with PD (8). Systematic reviews of interventions applied in older people with PD in stages 1–3 (9–11), certain therapies (such as yoga, pilates, hydro therapy, exergaming, balance, and gait training) have shown to improve motor function in folks with PD. In tests such as Unified Parkinson’s Disease Rating Scale (UPDRS-III), Berg Balance Scale (BBS), and Timed Up-and-Go (TUG), as well as HRQoL with the PD Quality of Life Questionnaire (PDQ-39 scale). These therapies have shown positive effects on physical and motor function parameters in older people with PD (9–11), there are novel alternatives such as Olympic combat sports (OCS) such as boxing (BOX), fencing, judo, karate, taekwondo, and wrestling, probably because of the stigma of risky activities (12), or else, because they are associated with a greater likelihood of injury in athletes (13). OCS (including BOX) can improve the health status of older people (14), leading to high energy expenditure due to the execution of intermittent high-intensity actions with multidirectional movements (15) that require predominantly an aerobic response during activity (13). In addition, the energetic contribution of combat sports is predominantly aerobic (16), specifically in BOX occupies 86%, followed by the glycolytic system (10%), and the ATP-PC system (4%) (17).

In a systematic review conducted by Valdés-Badilla et al. (18) in older people, significant improvements were reported in favor of OCS interventions in BBS and TUG tests regarding active/inactive control groups (CG). Similarly, Muñoz-Vásquez et al. (19), in a systematic review conducted in the non-athlete population, showed significant improvements in cardiorespiratory fitness measured with calorimetry in maximal oxygen consumption in favor of interventions with OCS versus CG active/inactive. In the systematic review conducted by Valdés-Badilla, Herrera-Valenzuela, Guzmán-Muñoz, et al. (20) in older people, significant improvements in HRQoL were found in favor of OCS interventions over CG. While interventions through the Rock Steady Boxing program modality have been something new and used in older people with PD (21), BOX is the most widely used OCS in exercise therapies or people with PD, and it has shown positive results in physical function parameters. It can help improve muscle strength, coordination, and balance, which is crucial for PD patients who often face mobility problems and fall risk (22). It also contributes to neuroplasticity, which could benefit patients’ cognitive function and mood (23). A systematic review by Chrysagis et al. (24) showed that BOX without one-on-one combat improved TUG, TUG dual task, ABC-Scale and PDQ-39 in older people with PD. In short, BOX without contact is beneficial in older people with PD.

Despite evidence indicating that combat sports with wrestling contact can cause neurological trauma, a meta-analysis reported that the practice of amateur, non-competitive BOX with headgear does not carry a significant risk of developing neurological dysfunctions (25). Early intervention in PD shows significant benefits, such as reduced symptoms, especially dyskinesia, and delayed initiation of levodopa treatment (26). Early treatment initiation in stages 1–3 can slow disease progression, directly impacting the patient’s HRQoL by reducing symptoms and potentially delaying serious complications (27).

Although there is evidence summarized in systematic reviews on the effects of BOX interventions in older people with PD (24). According to the Fau and Nabzo (28) and Papakostidis and Giannoudis (29), there is a knowledge gap on the findings that BOX is beneficial in older people with PD have not been evaluated by meta-analysis, stating that meta-analysis play a prominent role in producing high-quality evidence by increasing sample size and statistical power, which affirms that a meta-analysis on this topic could optimally guide clinical practice.

In this regard, this systematic review with meta-analysis aimed to evaluate the available body of published peer-reviewed studies on the effects of BOX interventions on balance, cardiorespiratory fitness, motor function, and HRQoL in older people with PD.

## 2 Methods

### 2.1 Protocol and registration

This systematic review followed the PRISMA guidelines (30). The protocol is registered with PROSPERO (the International Prospective Register of Systematic Reviews; ID code: CRD42024614097).

### 2.2 Eligibility criteria

The original, peer-reviewed papers published up until December 2024 that were not limited by language or publication date satisfied the inclusion requirements for this systematic review with meta-analysis. Conference abstracts, books and book chapters, editorials, letters to the editor, protocol records, reviews, case studies, and trials were among the resources that were not included. Furthermore, the PICOS (population, intervention, comparator, outcome, and study design) framework was employed in this systematic review (see Table 1).

**Table 1:** Selection criteria used in the systematic review.

Category	Inclusion criteria	Exclusion criteria
Population	Older people are considered as participants, with a mean age of 60 years or more, according to the World Health Organization (59), and without distinction of sex, who present PD. PD diagnosis is classified on the Hoehn and Yahr Scale in stages 1–3 (60).	Population under 60 years of age with PD and/or people over 60 years of age who do not have PD. People in advanced stages of PD (stage 4 or stage 5). People with a diagnosis of PD and other neurological diseases.
Intervention	BOX should have been the method of intervention in at least one of the study groups.	Interventions that do not use BOX. There are no details of the intervention procedure.
Comparator	Interventions with or without an active/inactive CG.	Observational studies (i.e., cross-sectional, retrospective, and prospective studies) that do not include structured comparison pre/post analysis.
Outcome	At least one assessment of physical function (i.e., UPDRS-part II and UPDRS-part III), such as balance and fall risk (i.e., dynamic and static balance, gait speed, walking speed, fall risk) or cardiorespiratory fitness (i.e., 6MWT, 10MWT, VO_2_max) and HRQoL (i.e., PDQ-39) before and after.	Lack of baseline data and/or follow-ups.
Study design	Experimental design studies (randomized and non-randomized controlled trials) with pre- and post-assessments.	Cross-sectional, retrospective, and prospective studies.

BOX, boxing; PD, Parkinson’s disease; UPDRS-part II, Unified Parkinson’s Disease Rating Scale two; UPDRS-part III, Unified Parkinson’s Disease Rating Scale three; HRQoL; health-related quality of life; PDQ-39, Quality of Life Questionnaire in PD.

### 2.3 Information search process and databases

Six generic databases were used in the search procedure, which took place between February 2024 and December 2024: Web of Science (core collection), PubMed, Medline, Psychology and Behavioral Sciences Collection (EBSCO), CINAHL Complete, and Scopus. Free language phrases pertaining to balance, motor function, cardiorespiratory fitness, BOX, and HRQoL in older people with PD were adopted by the US National Library of Medicine Medical Subject Headings (MeSH). The following search term was used: (“boxing” OR “boxings” OR “boxers”) AND (“physical function” OR “physical performance” OR “physical fitness” OR “functionality” OR “functional independence” OR “functional mobility” OR “health condition” OR “falls” OR “fall risk” OR “risk of fall” OR “balance” OR “static balance” OR “dynamic balance” OR “walking speed” OR “gait speed” OR “mobility” OR “cardiorespiratory fitness” OR “aerobic fitness” OR “aerobic capacity” OR “cardiovascular health” OR “maximum oxygen consumption” OR “VO2max” OR “VO2max”OR “VO2max” OR “VO2peak” OR “VO2peak” OR “VO2peak” OR “cardiorespiratory function”) AND (“QoL” OR “HRQoL” OR “quality of life” OR “quality of life perception” OR “health related quality of life” OR “health-related quality of life” OR “mental health” OR “psychological health” OR “body image perception” OR “life satisfaction” OR “lifestyle” OR “healthy lifestyle” OR “psychological well-being” OR “emotional well-being” OR “health status” OR “health status indicators” OR “vitality”) AND (“older adult” OR “older adults” OR “older people” OR “older subject” OR “aging” OR “aging” OR “aged”) AND (“Parkinson” OR “Parkinson disease” OR “Parkinson’s disease” OR “Neurodegenerative Diseases”). Two separate experts were consulted over the included articles and the inclusion and exclusion criteria in order to help find more pertinent studies. The experts had to meet two criteria: (i) possess a doctorate in sport science and (ii) have peer-reviewed papers on physical performance in different population groups and/or physical performance published in journals using Journal Citation Reports®'s impact factor. To prevent bias in their searches, we kept our search approach a secret from experts. Following these procedures, on December 30, 2024, we looked through a database for pertinent retractions or errata pertaining to the works on the list.

### 2.4 Studies selection and data collection process

The studies were exported using the EndNote reference manager (Version X9, Clarivate Analytics, Philadelphia, PA, USA). Separate searches were performed by JHM and ICC, who also removed duplicates and looked at abstracts and titles as well as full texts. Until now, no differences have been found. The process was repeated for searches inside reference lists and referrals from outside experts. After reviewing the texts of potentially appropriate papers, the justification for excluding those that did not meet the selection criteria was revealed.

### 2.5 Methodological quality assessment

The methodological quality of the selected studies was evaluated using TESTEX, a tool for exercise-based intervention studies (31). TESTEX scores were one possible exclusion criterion (31). There is a 15-point evaluation system (5 points for study quality and 10 points for reporting), according to Smart et al. (31), while a third author (THV) acted as a referee for cases that were on the borderline and required additional validation from another author (PVB), two authors (JHM and ICC) conducted this process independently.

### 2.6 Data synthesis

From the chosen studies, the following information was gathered and examined: (i) author and year of publication; (ii) country of origin; (iii) study design; (iv) sample’s initial and medication used; (v) number of intervention and CG participants; (vi) sample mean age; (vii) activities in the BOX and CG; (viii) training volume (total duration, weekly frequency, and time per session); (ix) training intensity; (x) HRQoL, balance, physical function, and cardiorespiratory fitness; and (xi) key findings of the studies.

### 2.7 Risk of bias in individual studies

Two independent investigators (JHM and ICC) assessed the risk of bias Version 1–2 (ROBINS-1 and RoB 2) of the included studies, and a third investigator (EVC) analyzed the results. For non-randomized controlled trials (NRCTs), ROBINS-1 was applied, while for randomized controlled trials (RCTs), RoB 2 was applied following the recommendations of the Cochrane Handbook for Systematic Reviews of Interventions for RCTs, which were the basis for this assessment (32, 33). The domains assessed in ROBINS-1 were bias due to confounding, bias due to selection of participants, bias in the classification of interventions, bias due to deviations from intended interventions, bias due to missing data, bias in the measurement of outcomes, and bias in the selection of the report result. While RoB 2 assessed the basis of the randomization procedure, deviations from planned interventions, missing outcome data, outcome assessment, and choice of reported outcome, the risk of bias was classified as “high,” “low,” or “some concerns.”

### 2.8 Summary measures for meta-analysis

Meta-analysis is part of the study’s methodology; PROSPERO (registration code: CRD42024614097) has all the details. Only when at least two articles were available were meta-analyses conducted in this instance (34). The pre-training and post-training mean and SD for each dependent variable were used to compute effect sizes (ES; Hedge’s g) for each balance attribute, motor function, cardiorespiratory fitness, and HRQoL in the BOX and CG. The change score SD was used to normalize the data. The 95% confidence intervals (95% CIs) are displayed with the ES values. The following scale was used to interpret the calculated ES: trivial: <0.2; small: 0.2–0.6; moderate: >0.6–1.2; large: >1.2–2.0; very large: >2.0–4.0; and extremely large: >4.0 (35). The random effects model was used to account for differences between studies that might affect the effect of BOX. Comprehensive Meta-analysis software (Version 2.0; Biostat, Englewood, NJ, USA). Statistical significance was set at *p* ≤ 0.05 (36) and was used to perform these calculations. In each trial, the random effects model (Der Simonian-Laird approach) was used to calculate and pool the SMD and MD of ABC-Scale, TUG, TUG dual task, 6-min walking test (6MWT), and PDQ-39 (BOX vs. CG). The fundamental premise of the random-effects model is that genuine effects (interventions, duration, among others) vary throughout studies and that samples are selected from populations with varying ES. The data were pooled if at least three studies showed the same results (37). Since PD is a progressive neurodegenerative disease, demonstrating slowing of disease progression may be important to compare between groups, not just looking for significant improvements in intervention versus CG (38).

Heterogeneity between trial results was tested with a Cochran’s *Q* test (23) and *I*^2^ statistic. *I*^2^ values of < 25%, 25–50%, and > 50% represent small, medium, and large amounts of inconsistency (39). Egger regression tests were performed to detect small study effects and possible publication bias (40).

### 2.9 Certainty of evidence

According to their evaluation of the GRADE scale, studies were classified as having high, moderate, low, or very low confidence (41). All analyses were initiated with a high degree of assurance because studies with both RCT and NRCT designs were included. If there were issues with bias, consistency, accuracy, precision, immediacy of results, or danger of publication bias, the analyses were downgraded (41). The studies were assessed independently by two writers (JHM, ICC), and any disputes were resolved by consensus with a third author (EVC).

## 3 Results

### 3.1 Study selection

**Figure 1:**
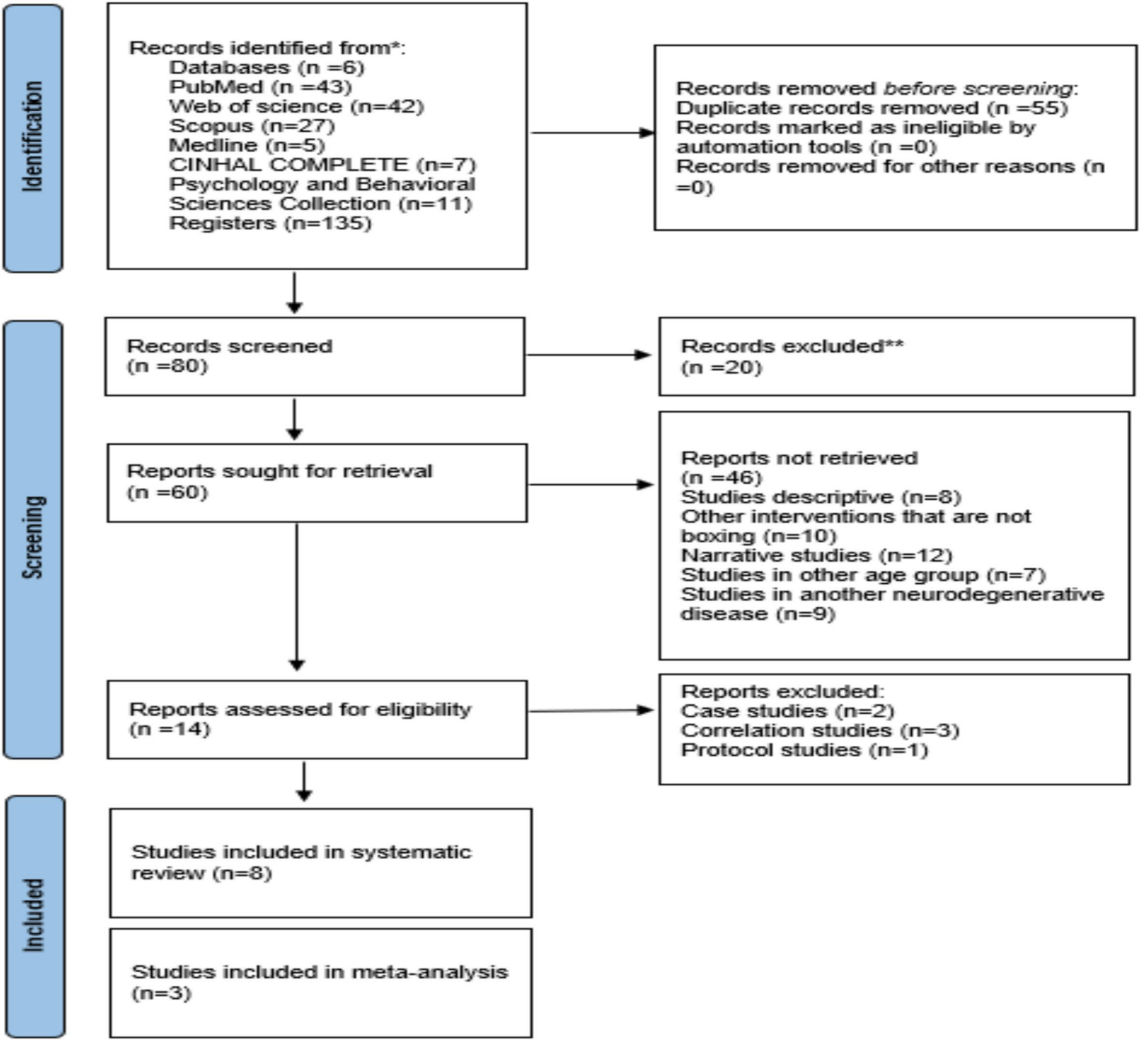
Adapted with permission from “PRISMA 2020 flow diagram template for systematic reviews” by Page et al. (30), licensed under CC BY 4.0.

Figure 1 details the search process for the studies. A total of 135 records were found. Subsequently, duplicates were eliminated, and the studies were filtered by selecting the title, abstract, and keywords, resulting in 80 references. In the subsequent analysis phase, 20 articles were excluded because the texts did not meet the search criteria, leaving 60. Subsequently, eight studies were descriptive, 10 other interventions not BOX, 12 narrative studies, seven studies in other age groups, and nine in another neurodegenerative disease. After this process, 14 potential studies remained, of which two were excluded case studies, three were correlation studies, and one was a protocol study. Therefore, eight studies met all selection criteria (42–49).

### 3.2 Methodological quality

The eight selected studies were analyzed using the TESTEX scale (Table 2). Only three studies achieved a score equal to or higher than 60% on the scale (42, 43, 46), while five studies did not achieve a score equal to or higher than 60% on the scale (44, 45, 47–49), namely 5/15 (45, 47, 49), 6/15 (44, 48), 11/15 (43, 46), and 12/15 (42).

**Table 2:** Study quality assessment according to the TESTEX scale.

Study	Eligibility criteria specified	Randomly allocated participants	Allocation concealed	Groups Similar at baseline	Assessors blinded	Outcome measures assessed >85% of participants *	Intention to treat analysis	Reporting of between group statistical comparisons	Point measures and measures of Variability reported **	Activity Monitoring in control Group	Relative exercise intensity reviewed	Exercise volume and energy expended	Overall TESTEX#
Combs et al. (42)	Yes	Yes	Yes	Yes	Yes	Yes (1)	Yes	Yes	Yes (1)	Yes	No	Yes	12/15
Domingos et al. (43)	Yes	Yes	No	Yes	Yes	Yes (2)	No	Yes	Yes (1)	Yes	Yes	Yes	11/15
Moore et al. (44)	Yes	No	No	Yes	No	Yes (1)	No	No	Yes (1)	No	Yes	Yes	6/15
Patel et al. (45)	Yes	No	No	Yes	No	Yes (1)	No	No	Yes (1)	No	No	Yes	5/15
Sangarapillai et al. (46)	Yes	Yes	Yes	Yes	Yes	Yes (1)	No	Yes	Yes (2)	Yes	Yes	Yes	12/15
Savoie et al. (47)	Yes	No	No	Yes	No	Yes (1)	No	No	Yes (1)	No	No	Yes	5/15
Shearin et al. (48)	Yes	No	No	Yes	No	Yes (1)	No	No	Yes (1)	No	Yes	Yes	6/15
Sonne et al. (49)	Yes	No	No	Yes	No	Yes (1)	No	No	Yes (1)	No	No	Yes	5/15

*Three points are possible: one point if adherence >85%, one point if adverse events were reported, and one point if exercise attendance was reported. **Two points possible: one point if the primary outcome is reported, one point if all other outcomes were reported. # total out of 15 points. TESTEX: Tool for assessing study quality and reporting in exercise.

### 3.3 Risk of bias within studies

**Figure 2:**
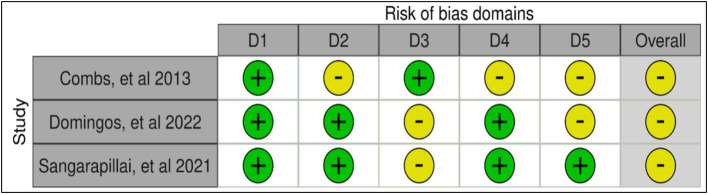
Risk of bias within studies. D1: randomization process; D2: deviations from the intended interventions; D3: missing outcome data; D4: measurement of the outcome; D5: selection of the reported result.

**Figure 3:**
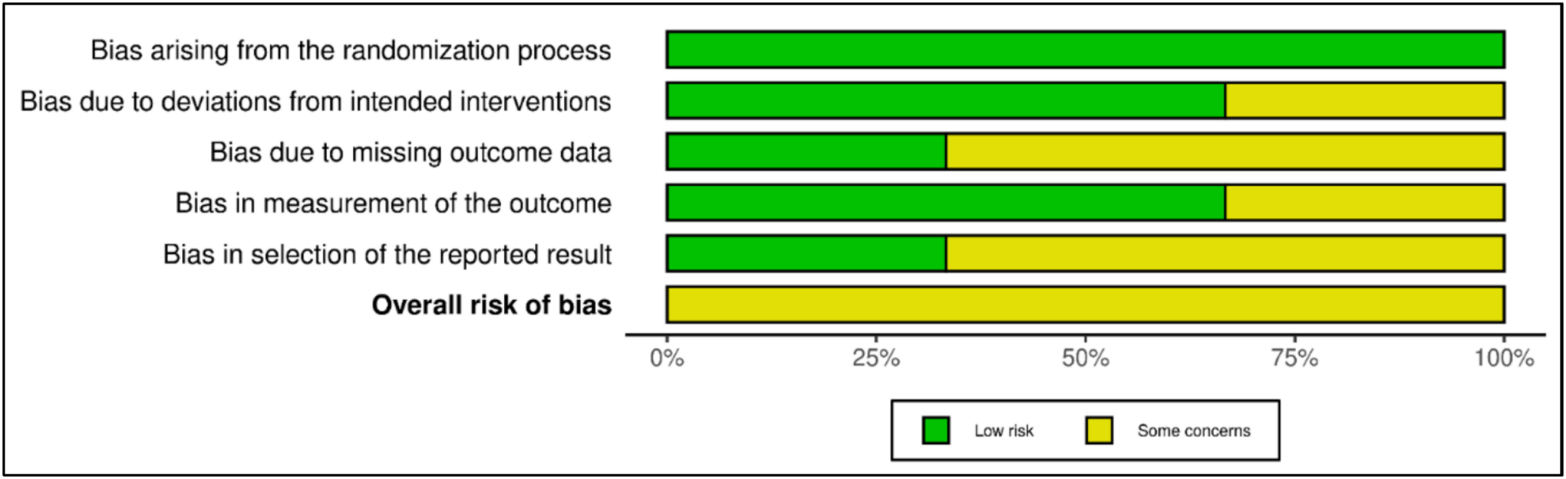
Risk of bias summary: review the authors’ judgments about each risk of a bias item in each included study.

The risk of bias was some concern in three studies (42, 43, 46), RCTs using RoB 2 for their analyses (33). In the randomization process, three studies showed low risk (42, 43, 46). While in deviations from the intended interventions, two studies showed low risk (43, 46), and one study showed some concerns (42). One study showed a low risk of missing outcome data (42), and two studies showed some concerns (43, 46). In measuring outcomes, two studies showed low risk (43, 46), and one study showed some concerns (42). In selecting the reported results, one study showed low risk (46), and two studies showed some concerns (42, 43). The risk of bias summary is presented in Figure 2, and the risk of bias graph is presented in Figure 3.

**Figure 4:**
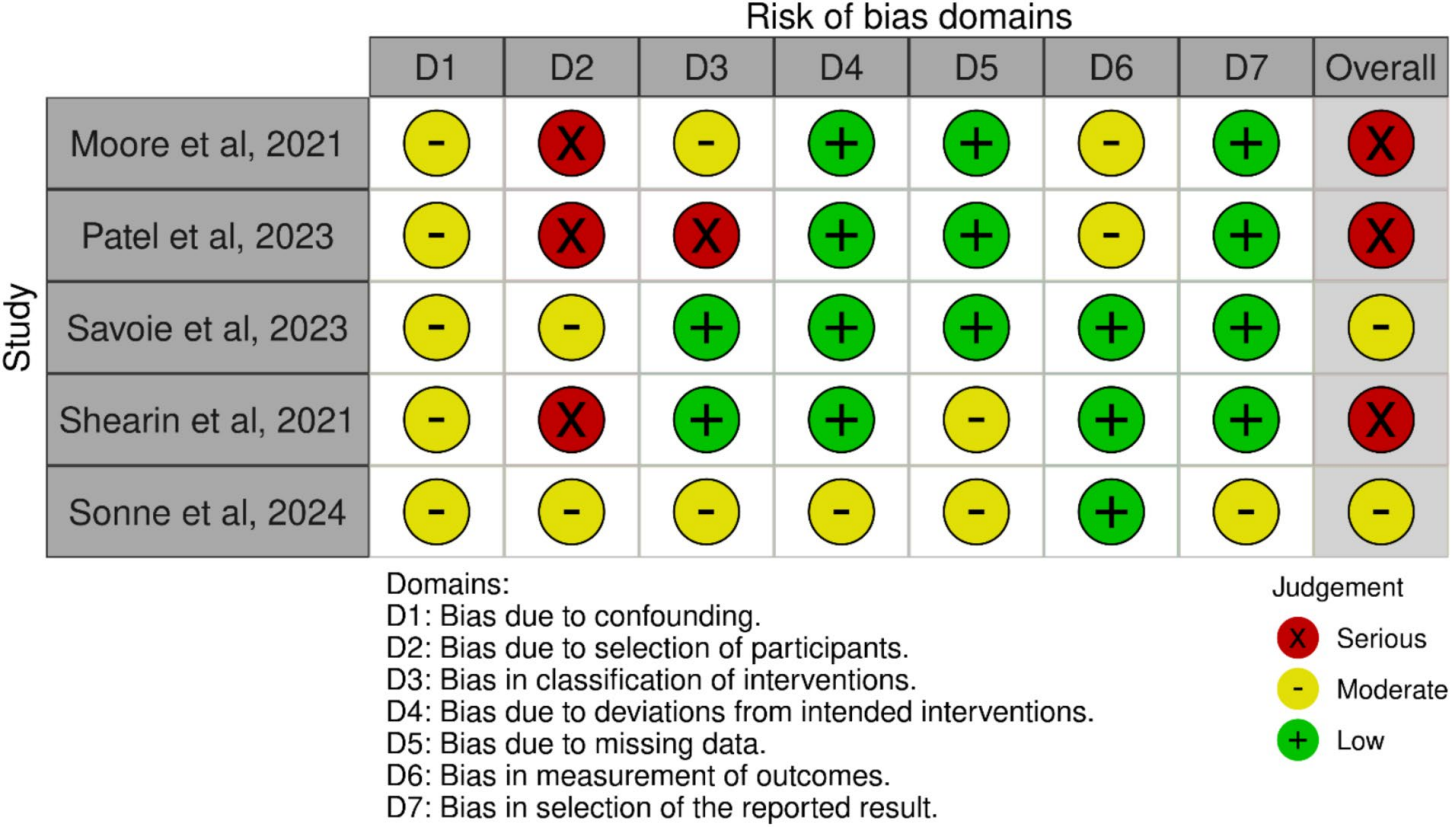
Risk of bias within studies.

**Figure 5:**
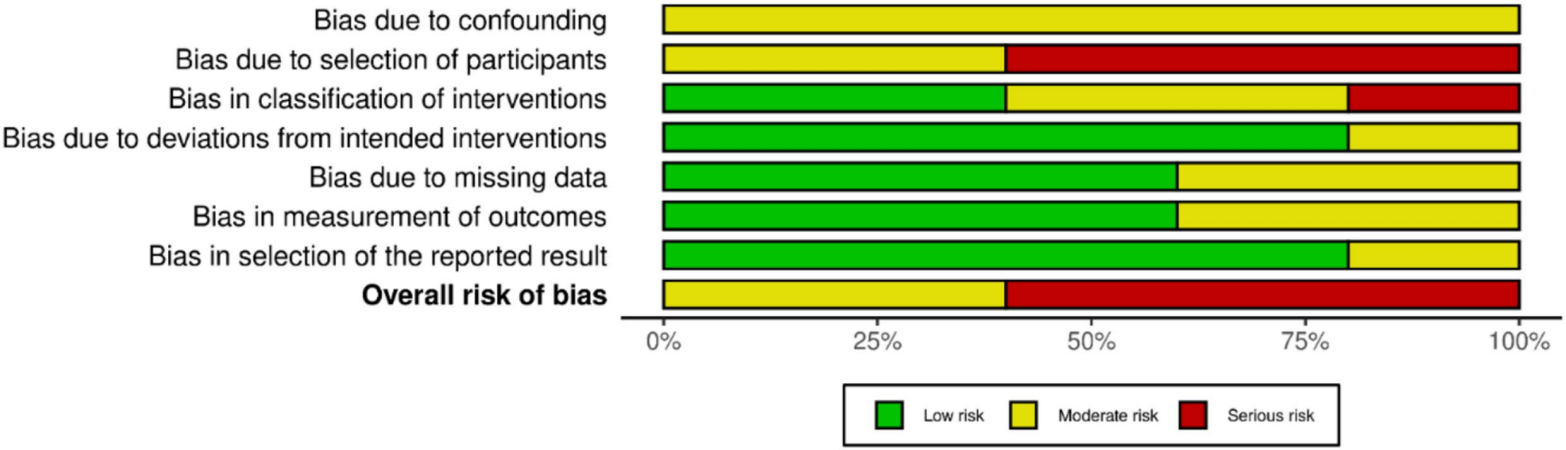
Risk of bias summary: review the authors’ judgments about each risk of a bias item in each included study.

All studies showed some concerns in the ROBINS-1 analysis in bias due to confounding (44, 45, 47–49). In the bias due to selection of participants, three studies showed high risk (44, 45, 48), and two studies showed some concerns (47, 49). In the bias in classification of interventions, two studies showed low risk (47, 48), two studies showed some concerns (44, 49), and one study showed high risk (45). In the bias due to deviations from intended interventions, four studies showed low risk (44, 45, 47, 48), and one study showed some concerns (49). In the bias due to missing data, three studies showed low risk (44, 45, 47), two studies showed some concerns (48, 49). In the bias in measuring outcomes, four studies showed low risk (44, 45, 47, 48), and one study showed some concerns (49). Finally, in bias in selection of the report result, two studies showed some concerns (47, 49), and three studies showed high risk (44, 45, 48). The risk of bias summary is presented in Figure 4, and the risk of bias graph is presented in Figure 5.

### 3.4 Studies characteristics

The variables analyzed in the eight selected studies are listed in Table 3. Four studies in the United States of America (42, 44, 45, 48), two in Canada (46, 47), one in the Netherlands (43), and one in the Multicenter (49).

**Table 3:** Studies report the boxing versus control group balance, cardiorespiratory fitness, on motor function, and quality of life in older people with Parkinson’s disease.

Study	Country	Study design	Sample’s initial and medication used	Mean (SD) since diagnosis	Groups (n)	Mean age (years)	Type of intervention and control group	Training volume			Training intensity	Balance and fall risk (assessments)	Cardiorespiratory fitness (assessments)	Motor function (assessments)	HRQoL (assessments)	Main outcomes
								Weeks	Frequency (sessions/week)	Session duration (minutes)						
Combs et al. (42)	United States	RCT	Subjects diagnosed with Parkinson’s Stages 1–3 NR	BOX: 41.5 ± 182.0 months CG: 50.0 ± 99.0 months	BOX: 17 (64% male and 36% female) CG: 14 (71% male and 29% female)	BOX: 66.5 ± 28.0 CG: 68.0 ± 31.0	BOX: adapted boxing CG: Multi-component training	12	3	90	NR	BBS (total) ABC-Scale (total) ABC-Scale (total)	Gait velocity (m/s) 6MWD (m)	NR	PDQ-39 (total)	**BOX vs. CG** **Both groups** ↑BBS ↑TUG dual task ↑TUG ↑PDQ-39 **BOX** ↑6MWD ↑Gait velocity **CG** ↑ ABC-Scale
Domingos et al. (43)	Netherlands	RCT	Subjects diagnosed with Parkinson’s Stages 1–3 NR	BOX: 6.10 ± 4.72 years CG: 9.09 ± 5.73 years	BOX:14 (60% male and 40% female) CG: 15 (73% male and 27% female)	BOX: 64.36 ± 11.14 CG: 63.69 ± 6.63	BOX: adapted boxing alone CG: Boxing with kicking	10	1	60	NR	ABC-Scale (total)-FES-I (total) ABC-Scale (total) TUG dual task (s) TUG (s)	6MWD (m)	NR	PDQ-39 (total)	**BOX vs. CG** **BOX** ↑ PDQ-39 ↑ TUG **Both group** ↔ TUG dual task ↔ ABC-Scale ↔6MWD ↔ Mini-BESTest
Sangarapillai et al. (46)	Canada	RCT	Subjects diagnosed with Parkinson’s Stages 1–3 Levodopa	BOX: 6.38 ± 4.9 years CG: 7.82 ± 5.2 years	BOX: 20 (reports no gender) CG: 20 (reports no gender)	BOX: 64.2 ± 9.8 CG: 65.1 ± 9.2	BOX: adapted boxing alone CG: sensory attention focused exercise	10	3	60	NR	NR	NR	UPDRS-III (score) CHAMPS (scores) Stride velocity (m/s) Stride length (m)	PDQ-39 (total)	**BOX vs. CG** **Both groups** ↑ PDQ-39 **CG** ↑UPDR-III ↑ Stride velocity ↑ Stride length
Moore et al. (44)	United States	NRCT	Subjects diagnosed with Parkinson’s Stages 1–3 NR	NR	BOX: 12 (75% male and 25% female) CG: no reported	BOX: 67.0 ± 6.1	BOX: adapted boxing	32	3	90	15–17 (RPE 20 points)	TUG (s) FAB score	NR	NR	NR	**BOX** ↑TUG ↑FAB
Patel et al. (45)	United States	NRCT	Subjects diagnosed with Parkinson’s Stages 1–3 Dopaminergic medication	7.9 ± 4.4 years	BOX: 14 (57% male and 43% female) CG: no reported	BOX: 62.2 ± 9.0	BOX: adapted boxing	12	2	60	NR	NR	NR	UPDRS-III (score) MDS-NMS (score)	PDQ-39 (total)	**BOX** ↑UPDRS-III ↑ MDS-NMS ↔ PDQ-39
Savoie et al. (47)	Canada	NRCT	Subjects diagnosed with Parkinson’s Stages 1–3 NR	4.87 ± 4.65 years	BOX: 26 (61% male and 39% female) CG: no reported	BOX: 69.0 ± 1.0	BOX: adapted boxing	64	2	90	NR	TUG (s) FAB score	NR	NR	PDQ-39 (total)	**BOX** ↑TUG ↔FAB ↑PDQ-39
Shearin et al. (48)	United States	NRCT	Subjects diagnosed with Parkinson’s Stages 1–3 NR	4.75 years	BOX: 26 (76% male and 24% female)	BOX: 68.38 ± 3.0	BOX: adapted boxing	12	2	60	4 to 7 (RPE 10 points)	NR	NR	**Self-selected gait parameters** **Fast gait parameters** **Backwards walking** **Dual task gait** [all assessments evaluated: velocity (cm/s); Stride length (cm); Cadence; Single and double support (%)]	NR	**BOX** **Self-selected gait parameters** ↑velocity ↑cadence **Fast gait parameters** ↔no parameters **Backwards walking** ↑cadence ↑ Stride length **Dual task gait** ↑velocity ↑ Stride length ↑single and double support
Sonne et al. (49)	Multicenter	NRCT	Subjects diagnosed with Parkinson’s Stages 1–3 NR	NR	BOX: 50 (79% male and 21% female) CG: no reported	BOX: 71.2 ± 8.56	BOX: adapted boxing	32	3	90	NR	TUG (s) FAB score	10MWT (m)	NR	NR	**BOX** ↑TUG ↑FAB ↔10MWT

BOX, boxing; CG, control group; NR, no reported; RCT, randomized controlled trial; NRCT, non-randomized controlled trial; BBS, Berg Balance Scale; ABC-Scale, Activities-Specific Balance Confidence Scale; TUG, Timed Up-And-Go; FAB, Fullerton Advanced Balance; 6MWT, 6-min walking test; 10MWT, 10-min walking test; RPE, Rating of Perceived Exertion; HRQoL, health-related quality of life; UPDRS-part II, Unified Parkinson’s Disease Rating Scale two; UPDRS-part III, Unified Parkinson’s Disease Rating Scale three; PDQ-39, Quality of Life Questionnaire in PD; FES-1, Falls Efficacy Scale International; MDS-NMS, The Movement Disorder Society Non-Motor Rating Scale.

Both groups = means that both groups improved; BOX= means that only the boxing group improved.(i) Self-selected gait parameters; (ii) Fast gait parameters; (iii) Backwards walking; (iv) Dual task gait = is the name of the test evaluated, being the indicators without bold the ones evaluated.

### 3.5 Sample characteristics

The eight studies selected had participants ranging from 12 to 50 (42–49). Consequently, the cumulative sample size in all these studies included 228 older people with PD, with a percentage greater than 50% of males with a mean age of 66 years (42–49). Study population ranged from 4.75 to 9.09 years with PD and stages 1–3 of the disease two studies did not report years of diagnosis (44, 49).

### 3.6 Dosing and conducted interventions

The interventions ranged from 10 weeks to 16 months, with 1 to 3 weekly sessions of 6,090 min (42–49). Regarding intensity, only two studies reported it measured with Rating of Perceived Exertion (RPE) with a range of 4–7 on the 10-point scale (48) and 15–17 on the 20-point scale (44).

The movements performed in all studies (42–49) were displacements, basic punches, and combinations, with the most performed punches (jab, hooks, and uppercuts). None of the studies analyzed performed one-on-one fights.

Regarding the CG, one study (42) conducted multi-component training (i.e., endurance, muscle strength, and balance), another study (43) conducted BOX exercises combined with non-contact kicking and punching, and one study (46) conducted sensory stimulation exercises (stretches, walking, and chair exercises where participants were encouraged to complete the exercises slowly, in a controlled manner and with their eyes closed).

### 3.7 Meta-analysis results

The overall effects of BOX on balance, cardiorespiratory fitness, and HRQoL are shown in Table 4. Forest plots are shown in Supplementary Figures S1S5 (Appendix 1, Supplementary Tables). There were small to large effect sizes (ES = −0.56 to 2.16), and no significant differences were reported (*p* > 0.05) in Activities-Specific Balance Confidence Scale (ABC-Scale), TUG, TUG dual task, 6MWD, and PDQ-39.

**Table 4:** Synthesis of the results of the studies included the effects of boxing on balance, cardiorespiratory fitness, and health-related quality of life in older people with Parkinson’s disease.

	*n* ^a^	ES (95% CI)	*p*-value	*I*^2^ (%)	Egger’s test (*p*)	RW (%)
Balance						
ABC-Scale (total)	2,2,2,58	−0.56 (−1.29 to 0.17)	0.13	55.8	**0.13**	6.31–8.04
TUG (s)	2,2,2,58	0.24 (−0.25 to 0.75)	0.34	52.2	**0.15**	6.74–7.15
TUG dual task (s)	2,2,2,58	0.20 (−0.29 to 0.71)	0.41	53.4	**0.18**	6.28–7.18
Cardiorespiratory fitness						
6MWD (m)	2,2,2,58	2.16 (−1.41 to 5.74)	0.23	0.00	**0.67**	6.74–7.62
HRQoL						
PDQ-39	3,3,3,98	−0.009 (−0.70 to 0.69)	0.98	72.2	0.00	3.93–5.16

Bolded *p*-values mean significant improvement (*p* < 0.05) in the experimental group after the boxing intervention compared to the control group, and (*p* > 0.05) represents a low risk of publication bias. ^a^Data indicate the number of studies that provided data for analysis, the number of experimental and control groups, and the total number of older people with PD included in the analysis.95% CI = 95% confidence interval; ES, effect sizes (Hedge’s g); HRQoL, health-related quality of life; RW, relative weight of each study in the analysis.

In motor function, there were significant improvements in the UPDRS-III test (*p* = 0.02) and MDS-NMS (*p* = 0.003) in the study of Patel et al. (45) employed a BOX intervention without a CG. However, in the study of Sangarapillai et al. (46), there were significant improvements in favor of CG (sensory attention-focused exercise) in UPDRS-III (*p* < 0.0001), stride velocity (*p* < 0.04), and stride length (*p* < 0.007) compared to a BOX intervention. However, in the Shearin et al.’s (48) study, there were significant improvements in self-selected gait parameters (velocity *p* = 0.04; cadence *p* = 0.02), backward walking (cadence *p* = 0.003; stride length *p* = 0.02), and dual-task gait (velocity *p* = 0.04; stride length *p* = 0.02; single and double support *p* = 0.03) in a BOX intervention with no CG. Although positive results favored BOX in motor function tests, this variable could not be meta-analyzed due to the variability of study designs.

### 3.8 Adverse events and adherence

None of the studies analyzed presented adverse events when performing the interventions with BOX (42–49). All studies showed adherence equal to or greater than 80%, with BOX interventions being supervised by certified instructors in all studies (42–49).

### 3.9 Certainty of evidence

The results of the certainty of evidence range from low to moderate and do not allow definitive recommendations to be made on using BOX interventions on balance, cardiorespiratory fitness, motor function, and HRQoL variables compared to CG in older people with PD (Table 5).

**Table 5:** GRADE assessment for the certainty of evidence.

Assessment of certainty							Number of patients		Effect		Certainty	Importance
Number of studies	Study design	Risk of bias	Inconsistency	Indirect evidence	Vagueness	Other considerations	[Intervention]	[Comparison]	Relative (95% CI)	Absolute (95% CI)		
Boxing versus sensory exercise for Parkinson’s disease: a double-blinded randomized controlled trial												
1	Randomized trials	Seriously ^to^	It’s not serious	It’s not serious	It’s not serious	None	20/40 (50.0%)	20/40 (50.0%)	Not estimable		⨁⨁⨁ ◯ Moderate ^to^	Important
Community-based group exercise for persons with Parkinson’s disease: a randomized controlled trial												
1	Randomized trials	Seriously ^to^	It’s not serious	It’s not serious	It’s not serious	None	14/31 (45.2%)	17/31 (54.8%)	Not estimable		⨁⨁⨁ ◯ Moderate ^to^	Important
Boxing with and without kicking techniques for people with Parkinson’s disease: an explorative pilot randomized controlled trial												
1	Randomized trials	Seriously ^to^	It’s not serious	It’s not serious	It’s not serious	None	14/29 (48.3%)	15/29 (51.73%)	Not estimable		⨁⨁⨁ ◯ Moderate ^to^	Important
A community-based boxing program is associated with improved balance in individuals with Parkinson’s disease												
1	Observational studies	Very serious ^b^	It’s not serious	It’s not serious	It’s not serious	None	12/12 (100.0%)		Not estimable		⨁⨁ ◯◯ Low ^b^	Important
A pilot study of a 12-week community-based boxing program for Parkinson’s disease												
1	Observational studies	Very serious ^b^	It’s not serious	It’s not serious	It’s not serious	None	14/14 (100.0%)		Not estimable		⨁⨁ ◯◯ Low ^b^	Important
Long-term impact of a community-based adapted boxing program on physical functioning and quality of life of individuals with Parkinson’s disease												
1	Observational studies	Seriously ^to^	It’s not serious	It’s not serious	It’s not serious	None	26/26 (100.0%)		Not estimable		⨁⨁⨁ ◯ Moderate ^to^	Important
The effect of a multi-modal boxing exercise program on cognitive locomotor tasks and gait in persons with Parkinson’s disease												
1	Observational studies	Very serious ^b^	It’s not serious	It’s not serious	It’s not serious	None	26/26 (100.0%)		Not estimable		⨁⨁ ◯◯ Low ^b^	Important
A retrospective analysis of group-based boxing exercise on measures of physical mobility in patients with Parkinson’s disease												
1	Observational studies	Seriously ^to^	It’s not serious	It’s not serious	It’s not serious	None	68/68 (100.0%)		Not estimable		⨁⨁⨁ ◯ Moderate ^to^	Important

^a^some concerns; ^b^high.

## 4 Discussion

### 4.1 Balance

In balance tests, specifically for the ABC-Scale, TUG, and TUG dual task, our meta-analysis did not report significant increases in favor of BOX compared to active/inactive CG. To our knowledge, this is the first meta-analysis conducted with BOX interventions in older people with PD. While the study by Domingos et al. (43) did not show significant improvements in the meta-analysis in balance tests, Combs et al. (42) reported significant increases after the implementation of the training program separately for the BOX group and the CG. Specifically, the CG that performed a traditional exercise program significantly improved the ABC-Scale, indicating improved balance confidence. The authors attributed this finding to the fact that the exercise program for the CG included dynamic and static balance exercises that simulated activities of daily living, while the BOX program did not include specific activities that challenged balance, unlike what was mentioned by Domingos et al. (43) who did not observe improvements in balance after the BOX intervention. In this regard, it is important to consider the duration and intensity of the interventions. For example, Combs et al. (42) performed 24 sessions of 90-min duration over 12 weeks. Domingos et al. (43) performed a total of 10 sessions with a duration of 60 min over 10 weeks. Performing only one training session per week may not be optimal for improving balance among older people with PD (42). On the other hand, neither intervention mentioned the intensity of their training, which is relevant to generating adaptations in the participants.

Regarding NRCT interventions, two studies presented significant improvements in balance using the TUG test through BOX interventions (44, 47). The TUG test assesses the time required for participants to get up from a chair and walk forward 3 meters, then turn around and backward, returning to the chair to sit down. In this sense, adapting BOX may have generated improvements in muscle strength through active movements in combat positions and punching techniques. In turn, improvements in the TUG test may be related to the techniques of executing punches that involve different foot movements generating force from the lower extremities, transferring the force to the middle area and then to the upper extremities to hit the target (44). This involves constant anticipatory adjustments that may have challenged the visual, somatosensory, and vestibular systems (50).

On the other hand, only one of the NRCT studies reported significant improvements for the Fullerton Advanced Balance (FAB) (44). This test assessed subtle and multidimensional changes in balance and fall risk in older people (51). In this sense, the physical demands of adapted BOX incorporate multi-modal exercises that require agility, strength, and aerobic capacity, which can challenge balance (24). Specifically, BOX sessions, through shadow BOX, speed and coordination exercises with punches to the bag involve dynamic weight shifting tasks in asymmetrical positions that may have favored improvements in the FAB test in the study by Moore et al. (44). Unlike the study by Savoie et al. (47), it did not report significant improvements in the FAB test. It is important to analyze the variables of the training programs to identify the possible causes of the discrepancies between the studies. However, the study by Savoie et al. (47) did not report the intensity of their training, unlike Moore et al. (44), who used an intensity based on 15–17 RPE (RPE 20 points).

Furthermore, possible differences between both studies may be attributed to FAB scores being relatively higher pre-intervention in Savoie et al. (47), so it may have been more difficult to achieve significant improvements. Specifically, the mean FAB score in the sample of Savoie et al. (47) was ~4 points higher than a comparable sample of individuals with PD. Although the duration of the intervention in Savoie et al. (47) was longer (16 months) than 6 months in the study by Moore et al. (44), not knowing the intensity at which the BOX training program was developed in Savoie et al. (47) makes further analysis difficult.

### 4.2 Cardiorespiratory fitness

In cardiorespiratory fitness, specifically for the 6MWT variable, our meta-analysis did not report significant increases in favor of BOX compared to active/inactive CG. This is similar to what was reported by Sonne et al. (49), who found no significant improvements in the 10MWT test for the BOX intervention. Regarding the two meta-analyzed studies, Domingos et al. (43) mentioned that the shorter duration and frequency of their intervention (10 weeks of training at a frequency of one time per week) may have limited improvements in the BOX group. In addition, the small sample size may have made it difficult to find significant differences between the groups. It is important to mention that while our meta-analysis for cardiorespiratory fitness did not report significant improvements for the 6MWT variable, in the meta-analyzed study by Combs et al. (42), they reported significant pre- and post-intervention improvements within the BOX group on the 6MWT test. These changes were insignificant between groups, and a medium ES between groups was only reported for the 6MWT test, reflecting that the magnitude of change in gait endurance after training was greater for the BOX group. These differences may be attributed to the circuit training protocol performed by the BOX group, as they were required to train as intensely as they tolerated and to increase repetitions during each 3-min training interval. This differs from the CG, in which participants were asked to train at a self-selected pace during the traditional training program. In addition, Sonne et al. (49) have suggested that to promote significant changes in gait speed testing, interventions should specifically target gait, being task-specific.

### 4.3 Motor function

Regarding motor function, this variable could not be meta-analyzed due to the variability of study designs. However, positive results favoring BOX in motor function have been reported. Patel et al. (45) reported significant improvements in the UPDRS-III and MDS-NMS tests through 12 weeks of training with a BOX program. In this regard, BOX has the benefit of incorporating multiple exercise modalities (aerobic exercise, strength training, balance, and footwork), which can lead to motor benefits in people with PD (24). In this sense, BOX practice also involves learning complex movements and combinations that generate a cognitive stimulus in participants (24). This added to the social aspects of practicing a sport in the community, which can enrich physical, social, and psychological aspects instead of doing less complex exercises such as walking or cycling.

On the other hand, Sangarapillai et al. (46) reported significant improvements in favor of the CG (sensory-focused exercise) for UPDRS-III, stride speed, and stride length compared to the BOX group. In addition, immediately after the intervention, the BOX program did not improve disease severity compared to the CG. Similar to what was reported in a study that assessed disease severity after a BOX program, improvements in only two of the six participants (52). The improvements in motor function by the CG that performed sensory-focused exercises may be attributed to the better functioning of dopaminergic neurons due to the increased sensory stimulation passing through the basal ganglia during exercise (46). In addition, sensory exercises can improve symptoms, such as stiffness and postural instability (53). Symptoms that can reduce forward propulsion during walking, affecting stride length and speed (46). In this sense, stiffness and postural instability in the CG may have translated into improvements in gait parameters compared to the BOX group, where participants continued with an altered gait. It is important to mention that none of the aforementioned studies reported the intensity of their training, a relevant variable to induce improvements and replicate training programs.

### 4.4 Health-related quality of life

For HRQoL, our meta-analysis did not report significant improvements in PDQ-39 (42, 43, 46), unlike what was reported by Savoie et al. (47), who reported significant increases in PDQ-39 scores. Specifically, they reported significant improvements in the dimensions of stigma and communication, concluding that BOX helps maintain HRQoL. However, it cannot nullify the impact of disease progression. It is important to mention that in the study by Savoie et al. (47), only the sample attended 58% of the biweekly sessions, so their results could underestimate the real effect of BOX on HRQoL in people with PD. On the other hand, regarding the meta-analyzed studies (42, 46) that compared a BOX training program with a traditional exercise program (multicomponent training and sensory training), reporting an improvement in self-perceived HRQoL independently of obtaining an improvement in disease severity in both groups at the end of the intervention. This can be attributed to participants’ improved physical fitness by exercising in a pleasant environment and feeling part of a community (54, 55). Although BOX is an individual combat sport, it has the particularity that it can be performed in a group in gyms, either for health or recreational purposes. This can provide a supportive environment and promote camaraderie by sharing with people with the same common goal (24, 45). In this context, participants share their concerns about disease progression and create a supportive environment during interventions. A recent systematic review reported that a sense of relatedness to others and a sense of competence and autonomy can develop in individuals, a sense of internal motivation, satisfaction, and well-being, leading to improved HRQoL (54, 55).

On the other hand, in another of the meta-analyzed articles, Domingos et al. (43) reported no significant differences between BOX and BOX plus kicking interventions (*p* = 0.46). However, a statistically significant difference was reported from the baseline to the final assessment for the BOX training group (*p* = 0.04). Based on the current literature, a higher training frequency of at least two times a week may be necessary to induce improvements in HRQoL variables in patients with PD (24). Furthermore, some studies that have analyzed the effects of dose suggest that more intense exercises bring greater benefits for people with PD (24); however, the intensity of the intervention was not mentioned in the study by Domingos et al. (43). Finally, Sangarapillai et al. (46) reported a significant difference in the group and time factors analysis. Specifically, the PDQ-39 for the BOX training group improved from 31.4 to 26.20 points after 10 weeks of intervention, and for the sensory training group, 35.33 to 30.62 points. Despite this, both groups had no significant group × time interaction effect. In the PDQ-39 questionnaire, the lower the score, the better HRQoL, indicating that both interventions improved their overall scores at the end of the training programs. According to Sangarapillai et al. (46), this may be explained by participants experiencing increased musculoskeletal gains through BOX rather than improvements in the underlying neurological disease. As mentioned above, it has been reported that during BOX interventions, participants have felt happy to be part of a group with a common goal (56). In this sense, BOX’s dynamic and fun nature may allow participants to feel part of a community, which may lead to a positive outlook on the severity of their disease. This is relevant, given that recent studies have suggested that perceived HRQoL may significantly affect a person’s well-being more than clinical outcomes. Therefore, these potential psychosocial benefits of group exercise warrant further investigation (57).

### 4.5 Dosage

The duration of the interventions varied from 10 weeks to 16 months, with one to three weekly sessions of 60–90 min (42–49). On the other hand, Chrysagis et al. (24) in a recent systematic review of BOX interventions in people with PD reported a duration in the studies of 10–12 weeks with a frequency ranging from 1 to 3 times per week with sessions of 60–90 min. Regarding intensity, only two studies in our systematic review reported RPE as a measure ranging from 4 to 7 on the 10-point scale (48) and 15–17 on the 20-point scale (44) unlike the systematic review by Chrysagis et al. (24) where the intensities of the BOX training programs were not reported. The American College of Sports Medicine has published certain recommendations for exercise prescription in people with PD (58). Suggesting a frequency of three to five times per week for aerobic training and two to three times per week for RCT training, flexibility, or balance training with a session duration of 20–60 min (58), which is in line with what was reported in the studies in our systematic review and meta-analysis.

### 4.6 Strengths and limitations

Our systematic review with meta-analysis presents the following limitations: (i) the lack of information on intensity in BOX training programs (only two studies mentioned it); (ii) the limited number of RCT studies (only three studies); (iii) the lack of long-term follow-up in studies that could determine the sustainability of BOX programs; (iv) the failure to perform a moderator analysis in order to make subgroup comparisons (e.g., the severity of PD), or by training dosage due to the low number of studies; (v) the low-to-moderate results of the certainty of evidence that does not allow definitive recommendations on the use of BOX interventions on balance, cardiorespiratory fitness and HRQoL in older people with PD; and (vi) the low methodological quality of the studies (only three studies scored 60% or higher). On the other hand, the strengths are as follows: (i) the methodological processes that followed the PRISMA, PROSPERO, TESTEX, RoB 2, and GRADE scales; (ii) the use of six databases: PubMed, Medline, EBSCO, CINAHL Complete, Scopus, and Web of Science (core collection); and (iii) that all meta-analyzed studies of physical performance showed a low risk of publication bias.

### 4.7 Practical applications

#### 4.7.1 Physical rehabilitation

BOX can be integrated into physical rehabilitation programs for older people with PD to potentially enhance motor function. However, the evidence for balance and cardiorespiratory fitness improvements is limited.

#### 4.7.2 Community-based programs

The high adherence rates suggest that BOX is a feasible and acceptable exercise intervention for this population, which can be implemented in community centers or gyms.

#### 4.7.3 Holistic approach

While BOX alone may not significantly improve balance or cardiorespiratory fitness, it can be part of a holistic approach that includes other forms of exercise, such as yoga or balance training, to address multiple aspects of health in PD patients.

### 4.8 Psychosocial benefits

#### 4.8.1 Community and support

BOX classes can foster community and support among participants, which may enhance their motivation and overall well-being.

#### 4.8.2 Mental health

Engaging in group BOX activities may improve mental health by reducing feelings of isolation and increasing social interaction.

### 4.9 Recommendations for future research

#### 4.9.1 Intensity and dosage

Future studies should focus on standardizing and reporting BOX interventions’ intensity and dosage to understand their impact better.

#### 4.9.2 Long-term effects

Research should aim to include long-term follow-up to determine the sustainability of benefits. Comprehensive Interventions: Investigating the effects of combining BOX with other therapeutic exercises could provide insights into more effective intervention strategies.

## 5 Conclusion

Individual results of BOX interventions report have a beneficial impact on physical fitness and HRQoL in older people with PD; however, our meta-analysis showed no significant changes in ABC-Scale, TUG, TUG dual task, 6MWD, and PDQ-39 compared to active/inactive controls. Nevertheless, due to the variability in training dosage, the type of BOX applied in the interventions, and the few RCT studies that do not allow for group analyses, a more critical analysis cannot be performed. Therefore, this is an emerging issue in this population, and more studies are needed to make definitive recommendations and to be able to implement this BOX intervention in clinical practice in older people with PD.

## Data Availability

The original contributions presented in the study are included in the article/Supplementary material, further inquiries can be directed to the corresponding author.
