# Supplementary material for: Effects of boxing interventions on physical fitness and health-related quality of life in older people with Parkinson’s disease: a systematic review with meta-analysis
Source: Front Public Health. 2025 Jun 6;13:1589512. doi: 10.3389/fpubh.2025.1589512 (PMC12179875; doi:10.3389/fpubh.2025.1589512)
Supplement: Supplementary file 1 [file Supplementary_file_1.docx]

**EFFECTS OF BOXING PROGRAM ON BALANCE, CARDIORESPIRATORY FITNESS, MOTOR FUNCTION AND QUALITY OF LIFE IN OLDER PEOPLE WITH PARKINSON'S DISEASE. A SYSTEMATIC REVIEW WITH META-ANALYSIS**

**Figure 1.** Forest plot of changes in ABC-Scale in older people with PD participating in boxing compared with older people with PD assigned as controls. Values shown are effect sizes (Hedges' g) with 95% confidence intervals (CI). The size of the squares plotted reflects the statistical weight of each study.

**Figure 2.** Forest plot of changes in TUG in older people with PD participating in boxing compared with older people with PD assigned as controls. Values shown are effect sizes (Hedges' g) with 95% confidence intervals (CI). The size of the squares plotted reflects the statistical weight of each study.

**Figure 3.** Forest plot of changes in TUG dual task in older people with PD participating in boxing compared with older people with PD assigned as controls. Values shown are effect sizes (Hedges' g) with 95% confidence intervals (CI). The size of the squares plotted reflects the statistical weight of each study.

**Figure 4.** Forest plot of changes in 6MWD in older people with PD participating in boxing compared with older people with PD assigned as controls. Values shown are effect sizes (Hedges' g) with 95% confidence intervals (CI). The size of the squares plotted reflects the statistical weight of each study.

**Figure 5.** Forest plot of changes in PDQ-39 in older people with PD participating in boxing compared with older people with PD assigned as controls. Values shown are effect sizes (Hedges' g) with 95% confidence intervals (CI). The size of the squares plotted reflects the statistical weight of each study.
